# Ruthenium nanoparticles decorated curl-like porous carbons for high performance supercapacitors

**DOI:** 10.1038/srep19949

**Published:** 2016-01-28

**Authors:** Bih-Show Lou, Pitchaimani Veerakumar, Shen-Ming Chen, Vediyappan Veeramani, Rajesh Madhu, Shang-Bin Liu

**Affiliations:** 1 Chemistry Division, Center for General Education, Chang Gung University, Taoyuan 33302, Taiwan; 2 Institute of Atomic and Molecular Sciences, Academia Sinica, Taipei 10617, Taiwan; 3 Department of Chemical Engineering and Biotechnology, National Taipei University of Technology, Taipei 10608, Taiwan; 4 Department of Chemistry, National Taiwan Normal University, Taipei 11677, Taiwan

## Abstract

The synthesis of highly dispersed and stable ruthenium nanoparticles (RuNPs; *ca*. 2–3 nm) on porous activated carbons derived from *Moringa Oleifera* fruit shells (MOC) is reported and were exploited for supercapacitor applications. The Ru/MOC composites so fabricated using the biowaste carbon source and ruthenium acetylacetonate as the co-feeding metal precursors were activated at elevated temperatures (600–900 ^o^C) in the presence of ZnCl_2_ as the pore generating and chemical activating agent. The as-prepared MOC carbonized at 900 ^o^C was found to possess a high specific surface area (2522 m^2^ g^−1^) and co-existing micro- and mesoporosities. Upon incorporating RuNPs, the Ru/MOC nanocomposites loaded with modest amount of metallic Ru (1.0–1.5 wt%) exhibit remarkable electrochemical and capacitive properties, achiving a maximum capacitance of 291 F g^−1^ at a current density of 1 A g^−1^ in 1.0 M H_2_SO_4_ electrolyte. These highly stable and durable Ru/MOC electrodes, which can be facily fabricated by the eco-friendly and cost-effective route, should have great potentials for practical applications in energy storage, biosensing, and catalysis.

Porous activated carbon (PAC)-related materials offer great advantages for practical applications^1^,^2^, such as adsorbents, energy storage, catalyst supports, and electrodes for fuel cells^3^. Moreover, owing to the intrinsic properties, such as high surface area, diversified morphology, good electrical conductivity, and tailorable porosity, PACs are also favorable materials as supports for metal nanoparticles (NPs) or metal oxides, and have been widely applied as biomolecule sensors^13^,^14^,^15^, biomedical engineering materials^16^, toxic molecules/heavy metal detectors^17^,^18^, and supercapacitors^18^,^19^,^20^,^21^,^22^,^23^,^24^,^25^,^26^,^27^,^28^,^29^,^30^,^31^,^32^,^33^. In terms of the latter, aside from the widely studied carbon materials such as graphene^34^,^35^,^36^,^37^,^38^, carbon nanotubes^37^,^38^,^39^,^40^, and ordered mesoporous carbons^41^,^42^,^43^,^44^, PACs are easy to prepare, eco-friendly, and cost-effective; they may be prepared from renewable biomass precursors through facile carbonization and activation routes, for examples, from tree barks or leaves^18^,^20^,^21^,^22^,^23^,^24^, plants^25^,^26^, fruits^27^,^28^, grain or seed shells^29^,^30^,^31^,^32^,^45^,^46^,^47^,^48^,^49^,^50^,^51^, lignin^52^,^53^, food derivatives^54^, marine products^33^, and so on. Typically, the synthesis of PACs from biomass feedstock invokes a chemical activation method in which activating agents such as ZnCl_2_, KOH, NaOH, or H_3_PO_4_ are commonly introduced along with the biomass precursor^2^,^14^,^15^,^20^,^32^. Upon completion of a subsequent carbonization treatment, the substrate was then washed with concentrated HCl to obtain the PAC as the final product^53^,^54^.

We report herein the synthesis of stable, highly dispersed ruthenium nanoparticles (RuNPs) on PACs derived from *Moringa Oleifera* fruit shells. *Moringa Oleifera* is a fast-growing, deciduous tree also known as the “drumstick tree”, mostly cultivated in Asian countries such as India, Philippines, and is also commonly seen in Africa, South America, the Caribbean and other Oceania countries^55^,^56^. Compare to other biomass feedstock, *Moringa Oleifera*, which belongs to the family *of Moringaceae*, is not only abundant in nature but also known to produe highly nutritious fruit^57^,^58^ that have medicinal^59^,^60^,^61^, and other applications^62^. Moreover, the *Moringa Oleifera* also contains cellulose, hemicellulose, and lignin that are desirable as precursors for fabrication of PACs with wormhole-like microstructures^62^,^63^,^64^,^65^,^66^,^67^ preferable as supports for electochemical and energy storage applicaions. By graphitizing the biowaste carbon precursor, namely *Moringa Oleifera* fruit shells along with ZnCl_2_ as activating agent under N_2_ atmosphere at elevated temperatures, the PAC (hereafter termed as *Moringa Oleifera* carbons; MOC-T_c_) so fabricated at different carbonization temperatures (T_c_ = 600–900 ^o^C) was further washed with HCl to remove the Zn^2+^ species. Subsequently, the as-synthesized MOC-900 was mixed with the metal precursor (*i.e.*, ruthenium(III) acetylacetonate; Ru(acac)_3_) in ethanol solution followed by a thermal reduction treatment to disperse RuNPs onto the carbon support. It is noteworthy that, here, the Ru(acac)_3_ serves as the metal precursor as well as a secondary carbon source, which warrants not only simultaneous reduction of Ru^III^ to Ru^0^ but also a high dispersion of RuNPs on the MOC^7^,^8^,^9^,^10^,^11^,^12^. As illustrated in Fig. 1, after a thermal reduction treatment at 900 ^o^C, the Ru_*x*_/MOC-900 nanocomposites so fabricated with vaired Ru loadings (*x* = 1.0 and 1.5 wt%) were found to exhibit excellent electrochemical properties desirable for electrochemical applications. To the best of our knowledge, this represents the first report to exploit *Moringa oleifera* fruit shells as the primary carbon source to fabricate PAC materials for such applications. As will be shown latter that the Ru/MOC nanocomposites prepared by this innovated facile and cost-effective route are indeed suitable as high-performance electrode materials for high-power supercapacitors.

## Results and Discussion

**Figure 2:**
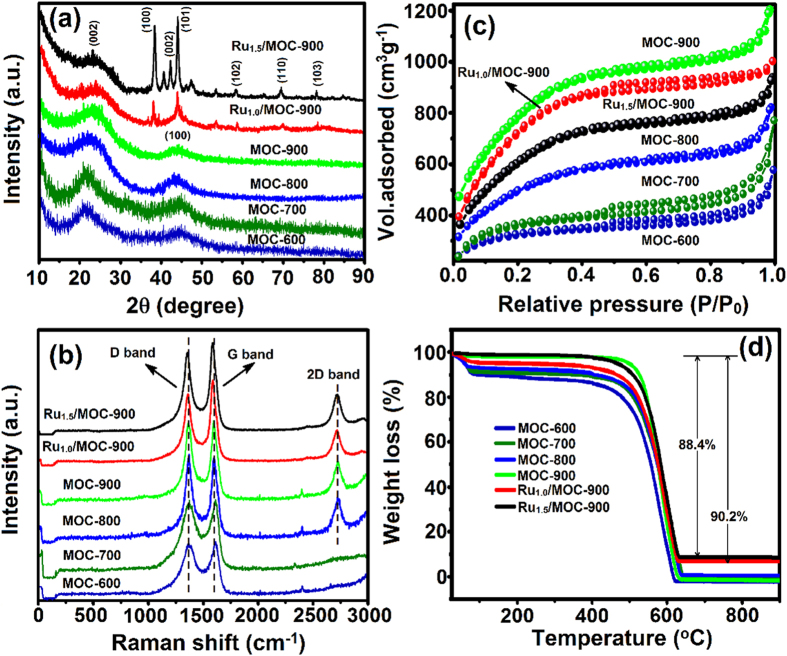
Physicochemical properties of various MOC-T_c_ and Ru_*x*_/MOC-T_c_ materials. (**a**) XRD profiles. (**b**) Raman spectra. (**c**) N_2_ adsorption/desorption isotherms. (**d**) TGA curves.

Figure 2a shows the XRD patterns of the as-prepared MOC and Ru/MOC samples. For the MOC-T_c_ samples prepared at different temperatures (T_c_ = 600–900 ^o^C), two broad peaks at 2θ = 22.2 and 43.4° respectively corresponding to the (002) and (100) diffractions of amorphous graphitic carbon^68^ were observed. On the other hand, for the Ru_*x*_/MOC-900 composites reduced at 900 ^o^C with different metal loading (*x* = 1.0 and 1.5 wt%), additional sharp diffraction peaks at 2θ = 38.3, 42.2, 44.0, 58.3, 69.4, and 78.4^o^ were evident, which may be assigned to the (100), (002), (101), (102), (110), and (103) diffraction planes of hexagonal close-packed (hcp) Ru metal (JCPDS-ICDD card No. 06-0663)^69^. The Raman spectra of the MOC and Ru/OMC samples exhibited two main peaks located at 1363 and 1585 cm^−1^ (Fig. 2b), which may be attributed to vibration bands of carbons in disordered graphite (D band) and the E_2_g mode of the graphite (G band), which is related to vibrations of sp^2^ carbon structure in two-dimensional (2D) hexagonal lattice^70^. Moreover, for MOC carbonized at elevated temperatures (T_c _≥ 800 ^o^C), an additional vibrational peak corresponding to the overtone of the D band (i.e., the 2D band) at 2712 cm^−1^ was also observed^71^. Accordingly, the G to D band intensity ratio (*I*_G_/*I*_D_) is normally used to assess the crystalline structure of the graphitic carbons, as depicted in Table 1. That an *I*_G_/*I*_D_ ratio of 1.21 was observed for the MOC-900 and Ru/MOC-900, indicating a high degree of graphitization for the PAC supports.

Results obtained from N_2_ adsorption/desorption isotherms (77 K) showed that all MOC-based samples exhibit H1-type isotherms (see Fig. 2c; *cf.* IUPAC classification). The presence of a weak hysteresis loop at *P*/*P*_0_ of *ca.* 0.4 together with the notable increase in N_2_ uptade at low relative pressures reveal the coexistence of micro- and mesoporosities in these carbon substrates^72^. Accordingly, the specific surface areas and pore volumes responsible for the micro- and mesoporosities may be derived, as depicted in Table 1. Further calculations by density functional theory (DFT) indicate that these MOC materials have an average mesopore size of *ca.* 4.0 nm (see Supporting Information, Fig. S1). Based on the above results, it is clear that the MOC-900, which possesses the highest BET surface area (*S*_tot_ = 2522 m^2^ g^−1^) and total pore volume (*V*_tot_ = 1.78 cm^3^ g^−1^), exhibits superior textural properties and *I*_G_/*I*_D_ value. Compared to its counterparts carbonized at relatively lower temperautres, it is indicative that the MOC-900 substrate is more suitable for application as electrode material owing to the anticipated higher electrical conductivity and porosity, which are favorable for electron transport and ion diffusion. Upon loading RuNPs onto the MOC-900 support, consistent decreases in both microporous and mesoporous surface area and pore volume with increasing Ru loading were observed (Table 1), indicating the successiful dispersion of metal NPs in both types of pores. This is also supported by the pore size distribution profiles (Supporting Information, Fig. S1), which showed notable decrease in micropores along with narrowing of mesopore distribution.

**Figure 3:**
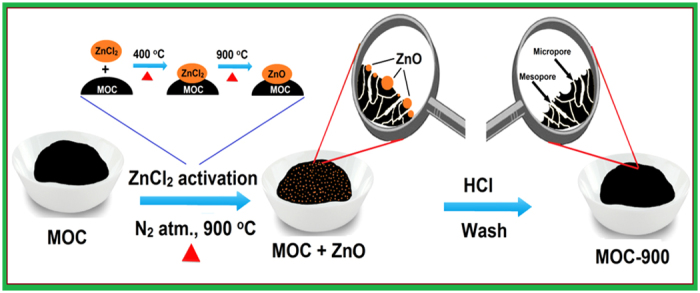
Schematic of the pore formation during activation of MOC at 900 ^o^C in the presence of ZnCl_2_ as activating agent.

The role of activating agent, namely ZnCl_2_, during activation of MOC is worthy for further exploration. During the process, the impregnated ZnCl_2_ tends to promote dehydration of the carbon substrate, leading to charring and aromatization along with the creation of porosities. It is anticipated that mobile liquid ZnCl_2_ (m.p. ∼ 283 ^o^C) should be formed during the initial stage of the activation. Further increasing the activation temperature beyond 750 ^o^C (b.p. of ZnCl_2_ *ca.* 730 ^o^C), strong interactions between carbon atoms and Zn species, which result in considerable collapses between the carbon interlayers to create pores in the matrix, as illustrated in Fig. 3 73. It has been shown that the generation of micro- and mesoporosities (Table 1) is provoked by the elimination of hydrogen and oxygen atoms from the carbon substrate by ZnCl_2_, leading to the formation of water rather than oxygenated organic species^74^. The amount of activating agent, activation temperature, and subsequent washing by HCl were all found to have profound effect on the evolution of porosity within the MOC. At a fixed activation temperature, a gradual increase in BET surface area (*S*_tot_) of the as-synthesized MOC with increasing dosage of ZnCl_2_ was observed (Supporting Information, Fig. S2). For examples, at a low carbonization temperature (600 ^o^C), the MOC prepared in absence of ZnCl_2_ exhibited a rather low *S*_tot_ = 50.6 m^2^ g^−1^. On the other hand, the surface area of MOC increased from *ca.* 210 to 718 m^2^ g^−1^ when prepared in the presence of 0.5 and 2.0 g of the activating agent (Supporting Information, Fig. S3). A more pronounced effect was found for activation temperature, for example, upon increasing the temperature from 600 to 900 ^o^C while in presence of a fixed amount of ZnCl_2_ (2.0 g), the surface area of the resulting MOC increased drastically from 718 to 2522 m^2^ g^−1^. These results are consistent with earlier literature reports^75^,^76^.

The TGA profiles of various MOC and Ru/MOC nanocomposites are displayed in Fig. 2d, their corresponding DTA curves are shown in Fig. S4 of the Supporting Information. Aside from the slight weight-loss below 150 ^o^C due to desorption of physisorbed water, all samples also showed a strong weight-loss at *ca.* 620 ^o^C, which should be associated with combustion of the MOC material^77^. The nearly complete weight-loss observed for the as-syntheized MOC-T_c_ (T_c_ = 600–900 ^o^C), indicating a complete oxidation of MOC by combustion and that nearly no trace of other ingredients (e.g., Zn species) were present in the PAC materials^78^. By comparison, while the Ru-loaded MOCs also exhibited two distinct weight-loss peaks at 50–150 and 400–620 ^o^C, respectively, a residual weight-loss of *ca.* 9.8 and 11.6 wt% was observed for the Ru_1.0_/MOC-900 and Ru_1.5_/MOC-900, respectively, indicating the anticipated presence of remanent rutheniumn oxides.

**Figure 4:**
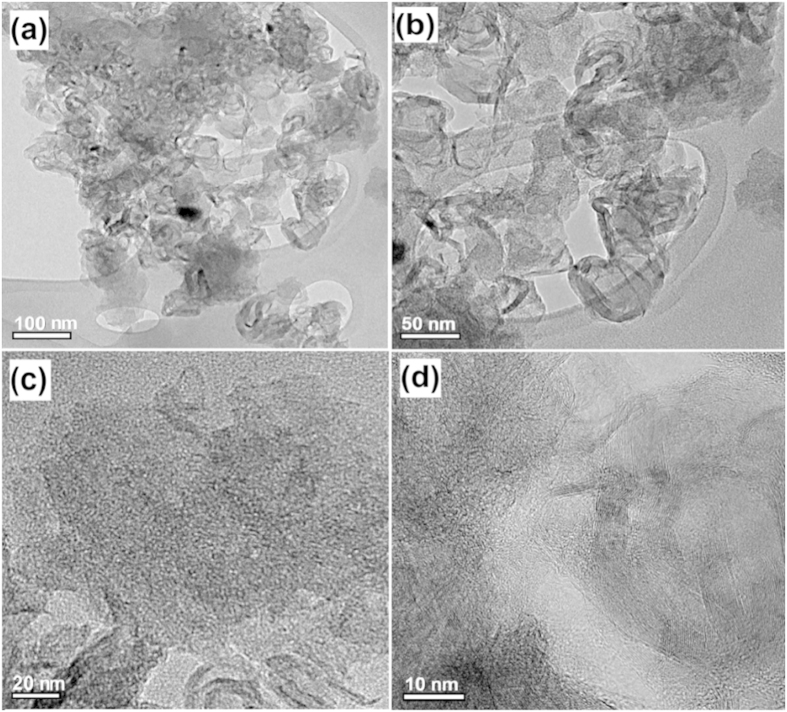
FE-TEM image of the MOC-900 at different magnifications. Scale bars: (**a**) 100, (**b**) 50 nm, (**c**) 20, and (**d**) 10 nm.

**Figure 5:**
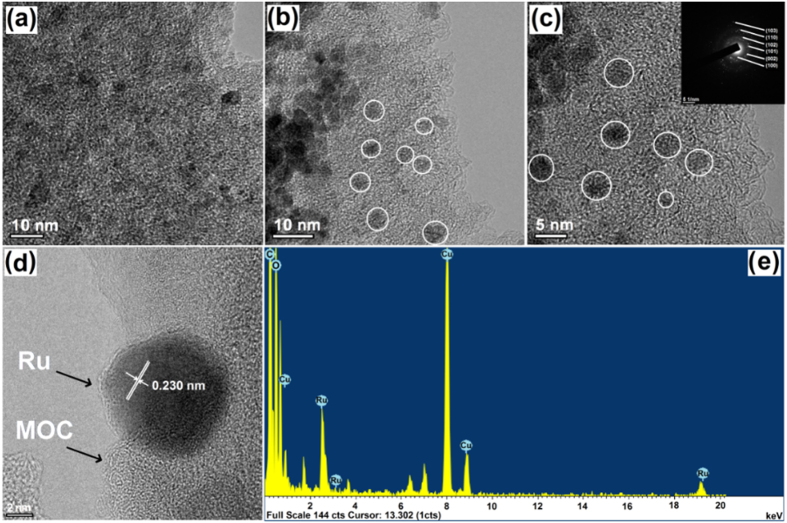
**(a–d)** FE-TEM images and (**e**) EDX profiles of Ru-loaded MOCs. (**a**) Ru_1.5_/MOC-900 and (**b–d**) Ru_1.0_/MOC-900 nanocomposites; the white circiles identify the presence of RuNPs. Inset in (**c**) shows the corresponding SAED pattern of RuNPs. (**e**) EDX profile of the Ru_1.0_/MOC-900 sample.

The morphology and structural properties of the as-syntheiszed and Ru-loaded MOCs are examined by using FE-SEM/TEM, as shown in Figs 4 and 5. The as-prepared MOCs clearly possess abundant porosities, which tend to increase with increasing carbonization temperature, as illustrated in the magnified SEM and TEM images (see Supporting Information, Figs. S5 and S6)^79^. This can be seen by the TEM images of MOC-900 sample taken at different magnifications (Fig. 4), which clearly indicate the presences of interconnected micro- and mesopores with a curl-like morphology. For the MOC-900, a rather board distribution of mesopore sizes in the range of *ca.* 5−20 nm on the surfaces of the curl-like MOCs may be inferred.

Moreover, the dispersion of RuNPs on the surfaces of MOC-900 may be clearly observed for both Ru-loaded samples, as shown in Fig. 5. Further analysis indicate that the RuNPs has an average particle size of ca. 3 nm for both Ru_1.5_/MOC-900 and Ru_1.0_/MOC-900 nanocomposites, as illustrated in Fig. S7 of the Supproting Information. For the latter, analysis based on selected area electron diffraction (SAED) pattern (Inset, Fig. 5c) revealed the presences of (100), (002), (101), (102), (110), and (103) reflections corresponding to crystalline hcp structure of Ru metal. Based on the FE-TEM image of a single Ru metal NP (Fig. 5d), a lattice spacing of 0.230 nm was determined, in excellent agreement with the value derived from the XRD data of the (002) lattice plane (Fig. 2a). Moreover, analysis of the energy-dispersive X-ray (EDX) result (Fig. 5e) indicates the existences of various signals corresponding to C Kα (0.2 keV), Cu Lα,β (0.9 keV), Cu Kα (8.0 keV), Cu Kβ (8.9 keV), Ru Lα,β (2.6 keV), Ru Lγ (3.2 keV), and Ru Kα (19.2 keV), respectively. The Cu signals arise from diffuse scattering of the Cu grid support^80^. The above results confirm a complete thermal reduction of Ru(acac)_3_ to RuNPs, which are uniformly dispersed over the structural framework of the MOC support.

**Figure 6:**
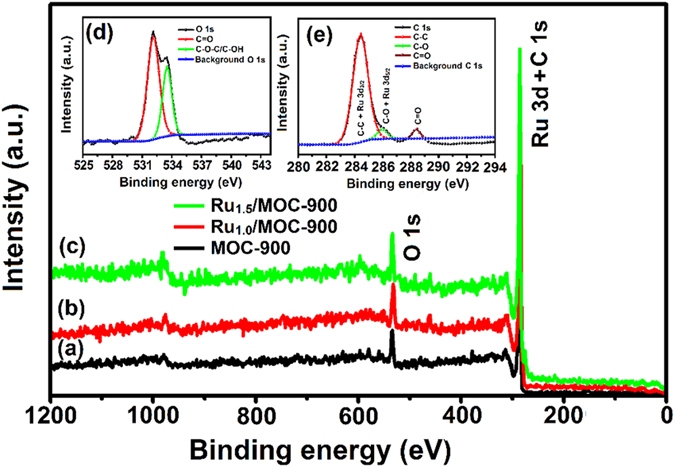
XPS spectra of various samples: (**a**) as-prepared MOC-900, (**b**) Ru_1.0_/MOC-900, and (**c**) Ru_1.5_/MOC-900 and their corresponding (**d**) C1s and (**e**) O1s spectrum.

The surface properties of the MOC-900 sample and Ru/MOC composites were further examined by X-ray photoelectron spectroscopy (XPS), as shown in Fig. 6. The XPS survey spectrum of the as-prepared MOC-900 (Fig. 6a) exhibits the anticipated C 1s (282–290 eV) and O1s (530–535 eV) signals. The spectrum near the C 1s and O 1s regions, which are displayed in Fig. 6d,e, respectively, may further be deconvoluted to identify C–C (284.8 eV), C–O (286.0 eV), and C = O (289.2 eV) functional groups as well as the corresponding oxygen states of C = O (521.8 eV) and C–O (533.1 eV). For the Ru_1.0_/MOC-900 and Ru_1.5_/MOC-900 nanocomposites, additional overlapping peaks at binding energy of 284.3 and 280.7 eV may be ascribed to the charistic peak of Ru 3d^3/2^ and Ru 3d^5/2^ 81, further confirming the formation of metallic Ru^0^ by the chemical reduction.

### Electrochemical Behavior of Ru/MOC-900 Electrodes

**Figure 7:**
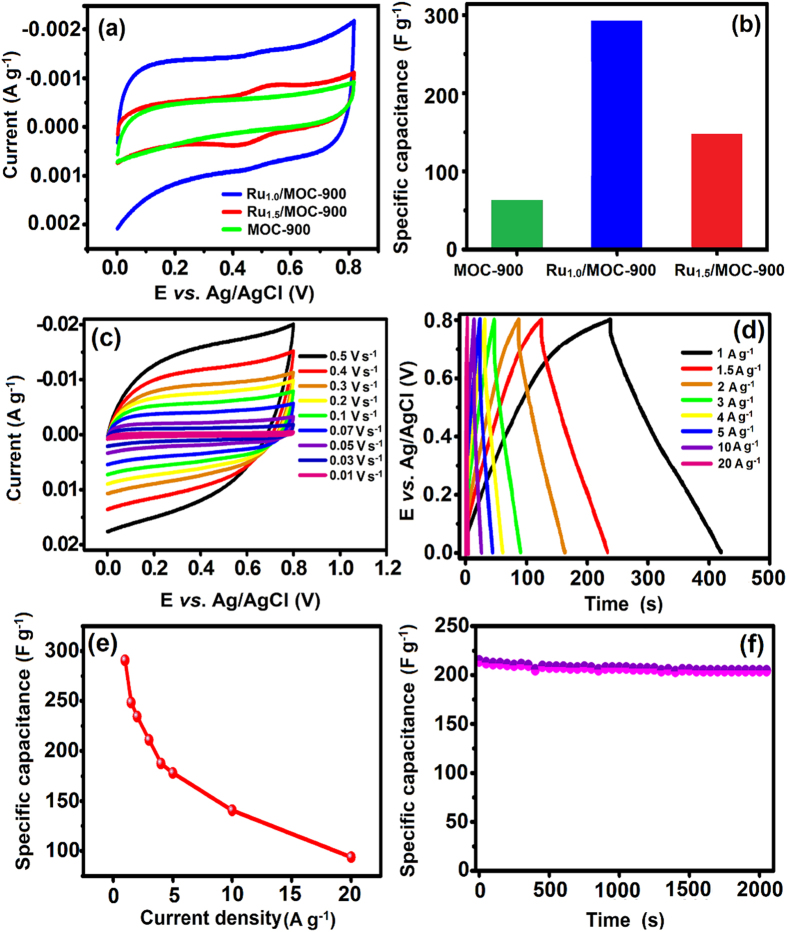
Electrochemical performances of assorted MOC-based electrodes. (**a**) CV curves recorded in 1.0 M H_2_SO_4_ aqueous electrolyte at a scan rate of 10 mV s^−1^. (**b**) Corrsponding specific capacitances observed for various electrodes. (**c**) CV curves recorded at different scan rates (10–500 mV s^−1^). (**d**) GCD curves at different current densities (1–20 A g^−1^). (**e**) Variations of specific capacitance with current density. (**f**) Cyclic stability test at a constant current density of 4 A g^−1^.

The electrochemical properties of the MOC-900, Ru_1.0_/MOC-900, and Ru_1.5_/MOC-900 electrode materials were assessed by cyclic voltammetry (CV) and galvanostatic charge-discharge (GCD) method. The CV curves observed for various Ru/MOC-based electrodes in 1.0 M H_2_SO_4_ aqueous electrolyte solution are depicted in Fig. 7a, which all exhibited the typical rectangular shape electri double-layer capacitor (EDLC) behavior. Nonetheless, compared to the Ru/MOC electrode, the metal-free MOC-900 electrode showed only weak capacitive behavior. Among the three MOC-based electrodes examined, the Ru_1.0_/MOC-900 electrode was found to exhibit an excellent pseudocapacitive redox property in the potential ranges of 0.4–0.6 V. As shown earlier, the RuNPs embedded in the Ru/OMC-900 are highly dispersed in the carbon support, leading to a notable decrease in total surface area and pore volume with increasing metal loading (Table 1). As such, the inferior capacitive performance observed for the Ru_1.5_/OMC-900 electrode compared to its counterpart with lower Ru loading (1.0 wt%) may be attributed to hindrance in electron transport and ion diffusion by the embedded metal. Overall, a maximum capacitance of 291 F g^−1^ was observed for the Ru_1.0_/MOC-900 electrode comparing to that of its MOC-900 and Ru_1.5_/MOC-900 counterparts, as shown in Fig. 7b.

To further assess the characteristics of the Ru_1.0_/MOC-900 nanocomposite for its practical application as electrode material for supercapacitors, we conducted further electrochemical studies. As can be seen from the CV curves recorded at vaired scan rates from 10 to 500 mV s^−1^ shown in Fig. 7c, all curves retained the rectangular-shape even at high scan rates, which indicates that the Ru_1.0_/MOC-900 electrode indeed exhibits excellent capacitive property with good electrical conductivity^82^. Moreover, based on the GCD curves measured for the Ru_1.0_/MOC-900 electrode at different current densities from 1 to 20 A g^−1^ (Fig. 7d), a gradual decrease in the corresponding specific capacitance with current densitiy from 291 to 94 F g^−1^ was observed (Fig. 7e). The durability of the Ru/MOC electrode was also evaluated. As shown in Fig. 7f, the Ru_1.0_/MOC-900 electrode retained over 90% of its initial capacitance after more than 2000 charge-discharge cycles when recorded at a constant current density of 4 A g^−1^, revealing an extraordinary stability and durability. For comparision, the textural and capacitive properties of other activated carbon (AC)-electrodes derived from various biomass feedstock are depicted in Table 2 23,24,28,45,46,47,48,49,50,51,52. It is indicative that the Ru/MOC electrode exhibits comparable capacitive performance even with a modest Ru loading of only 1 wt%. The MOC material derived from *Moringa Oleifera* fruit shells clearly has the advantage of achieving a high surface area (2473 m^2^ g^−1^), this together with the use of a activating agent (ZnCl_2_) and thermal reduction procedures help to facilitate dispersion of the metal (Ru) NPs and formation of micro- and mesoporosities favorable for electron transport and ion diffusion in the MOC matrix, hence, the superior electrochemical performances and excellent stability and durability well-suited for application of high-performance supercapactiors.

**Figure 8:**
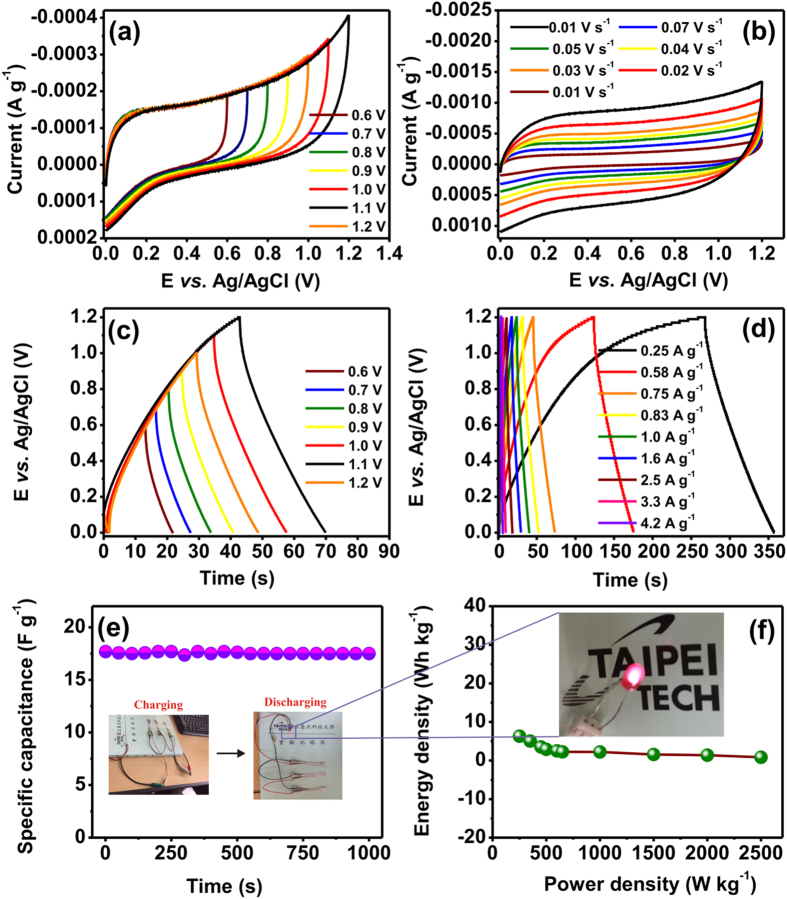
Performance assessments of the symmetric cell supercapacitor fabricated based on the Ru_1.0_/MOC-900 composite. CV curves recorded with varied (**a**) potential ranges and (**b**) scan rates. Corresponding GCD curves measured at varied (**c**) potential ranges and (**d**) current densities. (**e**) Cyclic stability test at a constant current density of 0.75 A g^−1^; insets: photographs of the as-prepared electrode during charge and discharge process. (**f**) Ragone plot of the solid-state device.

To enhance the energy densities, the fabricated symmetric cell supercapacitor was operated at various potential ranges (Fig. 8a) and scan rates (Fig. 8b). Accordingly, the corresponding calculated specific capacitance of 36. 2 to 11. 5 F g^−1^ was obtained using the known total mass of the electrode. The increase in capacitive current with increasing scan rate may be ascribed due to intercalation and deintercalation of ions^24^. Likewise, the GCD curves recorded at different potential ranges and current densities are displayed in Fig. 8c,d, respectively, which corresponds to a maximum specific capacitance and current density of 31.6 F g^−1^ and 0.25 A g^−1^, respectively. The specific capacitance so obtained is comparable to earlier literature reports^83^.

The long-term stability of the symmetric cell supercapacitor was also tested upto 1000 charge-discharge cycles at a current density of 0.75 A g^−1^. As a result, *ca.* 97% of its initial capacitance was retained after 1000 cycles (Fig. 8e). Finally, the correlation between power and energy densities of such solid-state device was also investigated; a maximum energy density of 6.3 Wh kg^−1^ at a power density of 250 W kg^−1^ was achieved (Fig. 8e).

In summary, a series of novel porous activated carbon materials derived from *Moringa Oleifera* fruit shells *via* a facile annealing and chemical activation process using ZnCl_2_ as activating agent have been developed and exploited as electrode support for electrochemical energy storage. When incorporated with RuNPs by thermal reduction using ruthenium(III) acetylacetonate as the metal precursor, the Ru/MOC materials so fabricated exhibits not only superior textural properties but also excellent capacitive properties with extraordinarly stability and durability. A maximum specific capacitance of 291 F g^−1^ was achieved for the Ru_1.0_/MOC-900 ^o^C electrode (Ru loading 1.0 wt%; activation temperature 900 ^o^C) at a current density of 1 A g^−1^. The results obtained from a cyclic charge-discharge test showed that the same electrode retained more than 90% of it original capacitance after 2000 consecutive test cycles at 4 A g^−1^. We believe that these MOC materials, which possess desirable textural properties, co-exsisting micro- and mesoporosities, and good electrical conductivities should render prospective applications in high-performance energy storage devices, biosensors, and catalysis, especially when combining with different metal oxides or conductive polymers as porous composites.

## Experimental

### Materials

Research grade ruthenium(III) acetylacetonate (Ru(acac)_3_, 96%; Acros), zinc chloride (ZnCl_2_; Sigma-Aldrich), potassium hydroxide (KOH; Sigma-Aldrich)) and poly vinyl alcohol (PVA; Shimakyu) were purchased commercially and used without further purification. Drumstick fruit shells (*Moringa Oleifera;* Family: *Moringaceae*) were collected from Theni district, Tamil Nadu, India. All solutions were prepared using doubly distilled water.

### Characterization Methods

All powdered x-ray diffraction (XRD) experiments were recorded on a PANalytical (X’Pert PRO) diffractometer using CuKα radiation (λ = 0.1541 nm). Nitrogen adsorption/desorption isotherm measurements were carried out on a Quantachrome Autosorb-1 volumetric adsorption analyzer at −196 ^o^C. Prior to measurement, the sample was purged with flowing N_2_ at 150 ^o^C for at least 12 h. The pore size distributions were derived from density functional theory (DFT) calculations. The morphology of the sample was studied by field-emission transmission electron microscopy (FE-TEM) at room temperature (25 ^o^C) using an electron microscope (JEOL JEM-2100F) operating at 200 kV. Elemental compositions of various samples were carried out with an energy-dispersive x-ray (EDX) analyser; an accessory of the FE-TEM facility. X-ray photoelectron spectroscopy (XPS) measurements were performed using a ULVAC-PHI PHI 5000 VersaProb apparatus. Thermogravimetric analyses (TGA) were done on a Netzsch TG-209 instrument under air atmosphere. All Raman spectra were recorded on a Jobin Yvon T64000 spectrometer equipped with a charge coupled device (CCD) detector cooled with liquid nitrogen. The backscattering signal was collected with a microscope using an Ar^+^ laser centered at 488 nm as the excitation source.

### Synthesis of Activated Carbon (MOC)

The *Moringa Oleifera* fruit shells were first air-dried and cut into pieces, then subjected to microwave irradiation (typically for 1–2 h) to obtain the pyrolytic MO fruit shells (denoted as MOFS). Typically, the activation of the carbon precursor was carried out by impregnating 1.0 g of MOFS in aqueous solution of ZnCl_2_ (2.0 g) under sonication, followed by an evaporation treatment at 80 ^o^C. The dried MOFS/ZnCl_2_ mixture was then heated at different temperatures (600, 700, 800, and 900 ^o^C) for 5 h under N_2_ atmosphere at a heating rate of 5 oC min^−1^. Finally, the sample was washed with 2M HCl solution and washed with hot deionized water until reaching a pH = 7. The sample was then dried at 80 ^o^C in an air oven. The final as-synthesized carbon products so obtained are named as activated *Moringa Oleifera* carbons (MOCs) and the samples prepared at different cabonization temperature (T_c_; in ^o^C) are denoted as MOC-T_c_.

### Preparation of Ru/MOC nanocomposite

Typically, a known amount of activated carbon (MOC) was first impregnated in nitric acid (HNO_3_) solution for 3 h at 80 ^o^C in an ultrasonic bath. The hydrophilic mixture was then filtrated and dried at room temperature. Then, 0.1 g of the treated powdered MOC-900 sample was impregnated into an ethanol solution (3 mL) containing desirable amount of Ru(acac)_3_ under continuous ultrasonication for 1 h. Subsequently, the sample vacuum dried to remove the solvent, then subjected to microwave irradiation (at 200 ^o^C for 2 h), followed by pyrolysis at high temperature (900 ^o^C) under N_2_ atmosphere for 5 h. The nanocomposites so prepared are denoted as Ru_*x*_/MOC-900, where *x* represents the Ru loading (*x* = 1.0 or 1.5 wt%).

### Fabrication of Ru_x_/MOC Electrodes

For supercapacitor applications, the Ru/MOC-electrodes were prepared by mixing Ru/MOC (85wt%) and graphite (15 wt%) with 0.4 mL of N-methylpyrrolidone to form a homogeneous slurry. Then, *ca.* 15 μL of the above slurry was coated on a stainless steel electrode with a dimension of about 1 × 1 cm^2^ by means of the solution-casting method, followed by drying overnight at 60 ^o^C. The mass loading of Ru/MOC on the substrate was estimated to be *ca.* 1.5 mg cm^−2^. For comparison purpose, separate MOC-electrodes (in absence of Ru) were also prepared following the above procedures. For fabricating the symmetric supercapacitor cell, two Ru/MOC electrodes were attached face to face by using PVA/H_2_SO_4_ gel electrolyte as a separator.

### Electrochemical Studies

Electrochemical properties of various MOC-900 and Ru_1.0_/MOC-900 electrodes were assessed by using a three-electrode system (in 1.0 M KOH aqueous electrolyte solution) consisting of the fabricated working electrode, Ag/AgCl as the reference electrode, and a platinum (Pt) wire as the counter electrode. All the cyclic votammetry (CV) and galvanostatic charge-discharge (GCD) experiments were performed on an electrochemical work station (CH Instrument; CHI627). Prior to each measurement, the cell was soaked in the aqueous electrolyte solution (1 M H_2_SO_4_) for a few hours. Typically, the CV profiles of the electrodes were recorded in the potential range of −1.0 to 0.0 V. The specific capacitance of various electrodes were calculated according to equation reported elsewhere^20^,^84^.

## Additional Information

**How to cite this article**: Lou, B.-S. *et al.* Ruthenium nanoparticles decorated curl-like porous carbons for high performance supercapacitors. *Sci. Rep.* **6**, 19949; doi: 10.1038/srep19949 (2016).

## Supplementary Material

Supporting Information

## Figures and Tables

**Figure 1:**
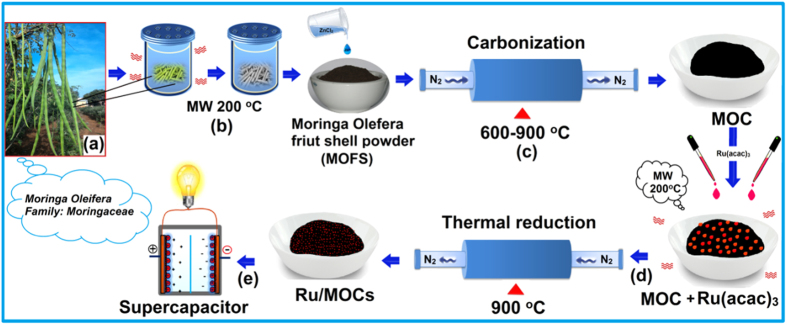
Illustration of the synthesis route for Ru/MOC nanocomposites. (**a**) *Moringa Oleifera* fruit shells; (**b**) thermal irradiation by microwave (MW); (**c**) activation and carbonization treatments; (**d**) addition of Ru(acac)_3_ followed by MW irradiation and thermal reduction at 900 °C; (**e**) Ru/MOC nanocomposite electrode for supercapacitor application.

**Table 1:** Textural properties of various as-prepared MOC and Ru/MOC samples.

Sample	T_c_^a^	Ru loading^b^	*M*_p_^c^	Surface area^d^		Pore volume^e^			*D*_p_^f^	*I*_G_/*I*_D_^g^
				*S*_tot_	*S*_micro_	*V*_tot_	*V*_micro_	*V*_meso_		
MOC-600	600	—	—	718	286	0.98	0.20	0.78	3.7	0.97
MOC-700	700	—	—	1384	456	1.02	0.30	0.72	3.8	0.98
MOC-800	800	—	—	1924	480	1.33	0.39	0.94	3.9	0.99
MOC-900	900	—	—	2522	576	1.78	0.46	1.33	4.3	1.21
Ru_1.0_/MOC-900	900	1.0	2.8	2473	506	1.69	0.37	1.32	4.1	1.20
Ru_1.5_/MOC-900	900	1.5	3.1	2015	439	1.48	0.29	1.19	3.9	1.21

(**a**) Carbonization temperature in ^o^C. (**b**) Ru loading in wt% deduced from EDX and TGA results. (**c**) RuNP size in nm determined by FE-TEM analysis. (**d**) Brunauer-Emmet-Teller (BET) surface areas in unit of m^2^ g^−1^; *S*_tot_ and *S*_micro_ denotes total and microporous surface area, respectively; *S*_micro_ determined by t-plot analysis. (**e**) Total pore volume in cm^3^ g^−1^ calculated at P/P_0_ = 0.99 of the N2 adsorption/desorption isotherm; *V*_tot_, *V*_micro_, and *V*_meso_ represents total, microporous, and mesoporous pore volume, respectively, *V*_meso_ = *V*_tot_–*V*_micro_. (**f**) Average pore size determined by non-local DFT calculations. (**g**) G to D band intensity ratio obtained from Raman data.

**Table 2:** Comparisons of textual and capacitive properties of various biomass-derived activated carbons.

Biomass feedstock	*S*_BET_^a^	Precursor/ZnCl_2_^b^	C^c^	Electrolyte	Reference
Sugar cane bagasses	1000	1:1.75	300	1 M H_2_SO_4_	23
Banana fibers	1097	1:5	296	1 M Na_2_SO_4_	28
Coffee beans	1019	1:1	368	1 M H_2_SO_4_	45
Coffee shells	842	1:20	150	6 M KOH	46
*Camellia Oleifera* fruit shells	1935	1:4	374	1 M H_2_SO_4_	47
Walnut shells	1073	2 M ZnCl_2_	117	6 M KOH	48
Fermented rice	2106	—	219	0.1 M KOH	49
Potato wastes	1052	1:2	255	2 M KOH	50
Coconut shells	2440	1:3	246	0.5 M H_2_SO_4_	51
Waste filter paper	2170	1:4	302	6 M KOH	52
*Moringa Oleifera* fruit shells	2473	1:1	291	1 M H_2_SO_4_	This work

(**a**) BET surface area in m^2^ g^−1^. (**b**) Ratio of carbon precursor to ZnCl_2_. (**c**) Capacitance in F g^−1^.
