# Supplementary material for: Ruthenium nanoparticles decorated curl-like porous carbons for high performance supercapacitors
Source: Sci Rep. 2016 Jan 28;6:19949. doi: 10.1038/srep19949 (PMC4730158; doi:10.1038/srep19949)
Supplement: Supporting Information [file srep19949-s1.pdf]

Supporting Information

Ruthenium nanoparticles decorated curl-like porous carbons for high performance supercapacitors

Bih-Show Lou[1*], Pitchaimani Veerakumar[2], Shen-Ming Chen[3,*], Vediyappan Veeramani[3], Rajesh Madhu[3] & Shang-Bin Liu[2,4,*]

1Chemistry Division, Center for General Education, Chang Gung University, Taoyuan 33302, Taiwan. 2Institute of Atomic and Molecular Sciences, Academia Sinica, Taipei 10617, Taiwan. 3Department of Chemical Engineering and Biotechnology, National Taipei University of Technology, Taipei 10608, Taiwan. 4Department of Chemistry, National Taiwan Normal University, Taipei 11677, Taiwan.

Correspondence and requests for materials should be addressed to S.B.L. (email: sbliu@sinica.edu.tw) B.S.L. (email: blou@mail.cgu.edu.tw) or S.M.C. (email: smchen78@ms15.hinet.net).

****

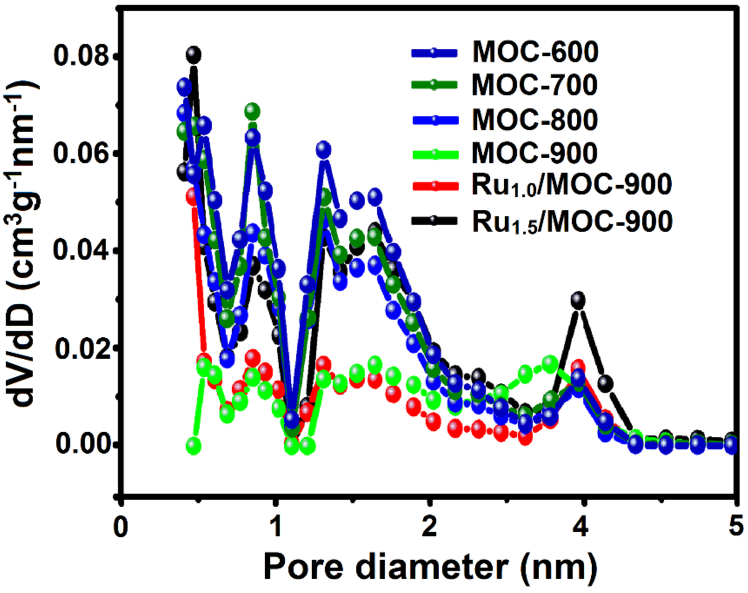

Figure S1. DFT pore size distributions of various as-synthesized MOCs and Ru/MOC-900 nanocomposites.

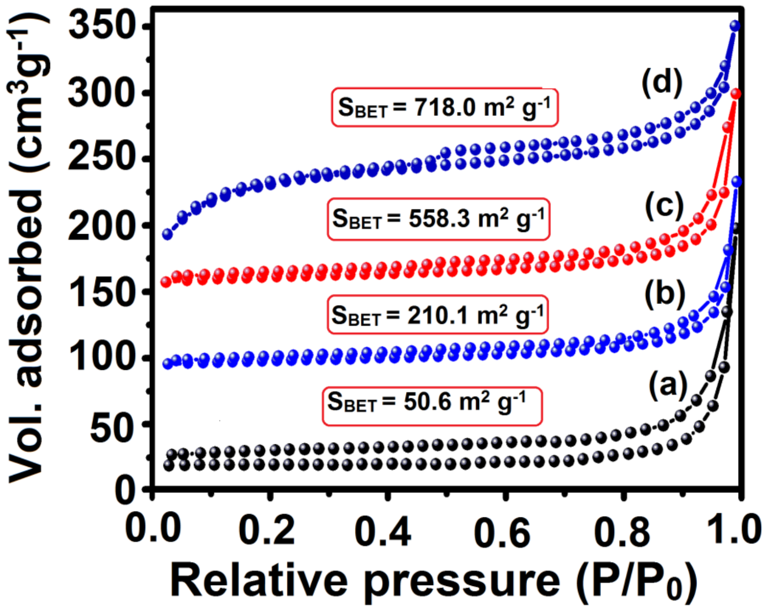

Figure S2. N_2 adsorption/desorption isotherms of the porous activated carbons prepared from biomass precursor (*Moringa Oleifera* fruit shells) carbonized at 600 ^{o}C under N_2 atmosphere (a) without, and (b–d) with the presence different amounts of $ZnCl_2$ activating agent: (b) 0.5 g, (c) 1.0 g, and (d) 2.0 g.

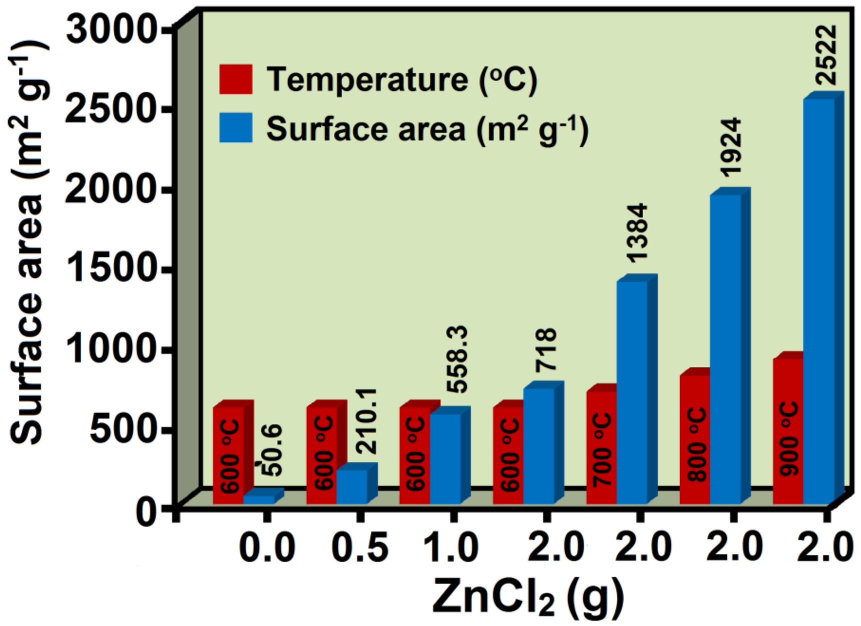

Figure S3. Effects of $ZnCl_2$ and carbonization temperature on surface areas of the resultant MOCs.

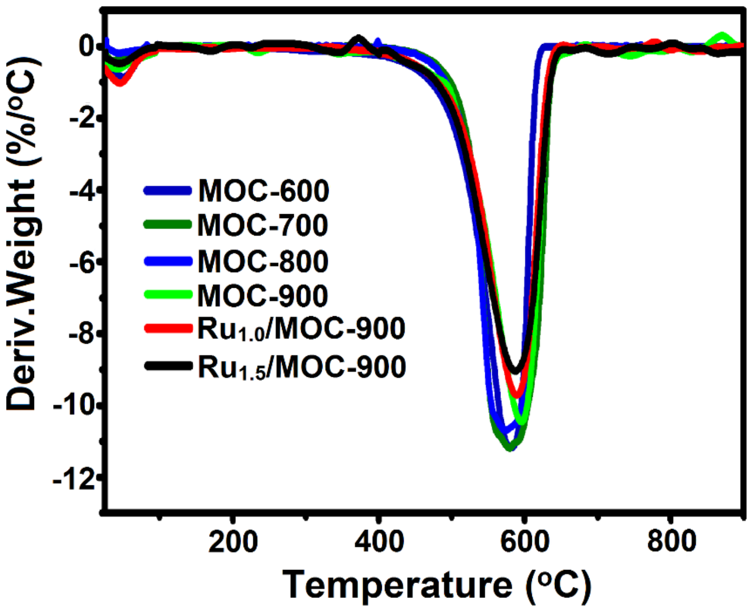

Figure S4. DTA curves of various as-synthesized MOCs and Ru/MOC-900 nanocomposites.

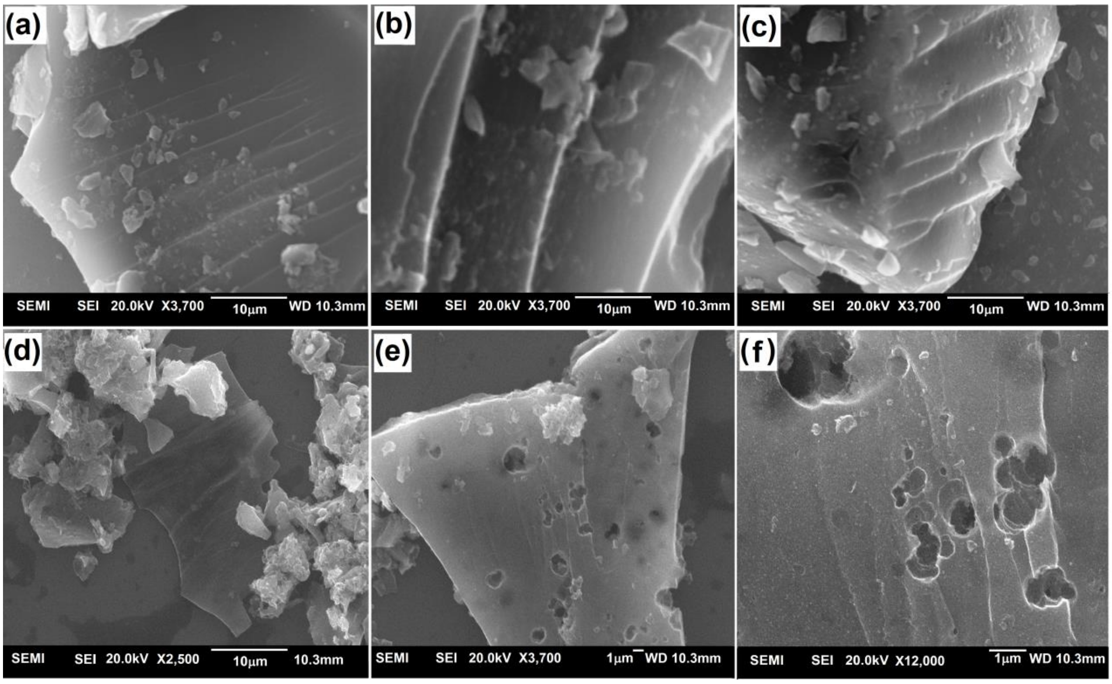

Figure S5. FE-SEM images of the as-synthesized (a) MOC-600, (b) MOC-700, (c) MOC-800, and (d–f) MOC-900 samples at various magnifications.

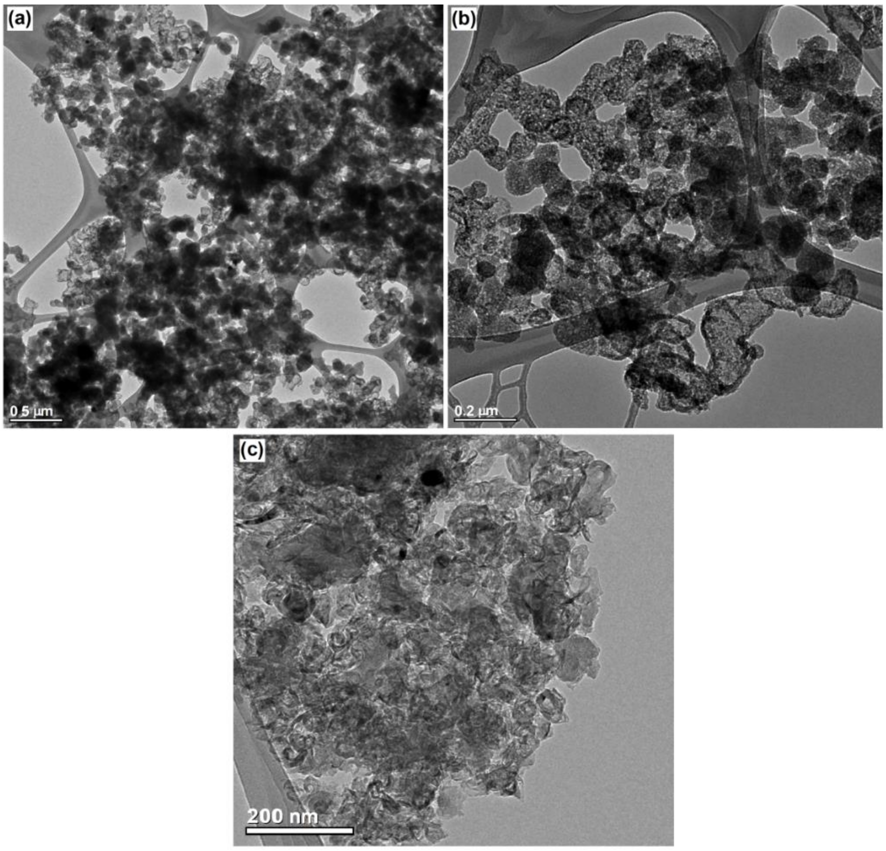

Figure S6. FE-TEM images of the as-synthesized (a) MOC-600, (b) MOC-700, and (c) MOC-800 samples.

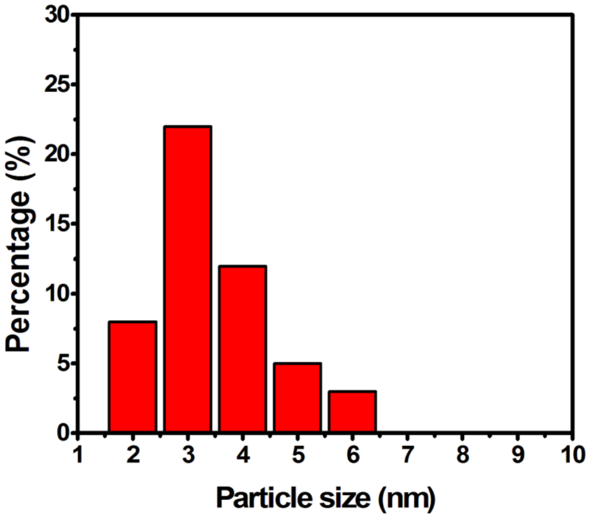

Figure S7. Particle size distribution of RuNPs for the $Ru_{1.0}$/MOC-900 nanocomposite.
